# Case Report: Transitional meningioma in a free ranging mantled howler monkey (*Alouatta palliata*)

**DOI:** 10.3389/fvets.2026.1857938

**Published:** 2026-08-14

**Authors:** Mario Romero-Vega, Katharina Gregor, Isabel Hagnauer, Alejandro Alfaro-Alarcón, Andreas Beineke

**Affiliations:** 1 Department of Pathology, University of Veterinary Medicine Hannover, Foundation, Hannover, Germany; 2 Department of Pathology, School of Veterinary Medicine, National University, Heredia, Costa Rica; 3 Rescate Wildlife Rescue Center, Fundación Restauración de la Naturaleza, Alajuela, Costa Rica; 4 Berlin Institute of Health, Institute of Virology, Charité-Universitätsmedizin, Berlin, Germany

**Keywords:** cancer, neurology, pathology, primate, veterinary

## Abstract

Neoplasia in free ranging wildlife remains poorly documented, particularly in non-human primates. An adult free ranging female mantled howler monkey (*Alouatta palliata*) was found conscious but non-ambulatory on a country road in northwestern Costa Rica. Following admission to a wildlife rescue center, euthanasia was elected due to poor prognosis and body condition. At necropsy, three firm, gritty, white masses were identified on the inner surface of the parietal bones. Histopathologic examination revealed a well-circumscribed, moderately cellular, encapsulated neoplastic proliferation arising from the dura mater and compressing the cerebral cortex while sparing the leptomeninges. Neoplastic cells were arranged in whorls and occasional interlacing streams, with sporadic psammoma bodies. A diagnosis of transitional meningioma was made. Immunohistochemically, neoplastic cells were positive for S100 and negative for pan-cytokeratin, vimentin, and E-cadherin. Reports of meningiomas in non-human primates, particularly in free ranging individuals, are scarce. This case contributes to the limited knowledge of neoplasia in wildlife and underscores the value of documenting neoplasia in free ranging primates.

## Introduction

Neoplasia in wildlife populations remains an under-researched topic due to several limitations that hinder a comprehensive study (1). These challenges include difficulties in tracking individual animals, performing necropsies under field conditions, rapid decomposition in sylvatic landscapes, and the overall lack of systematic disease surveillance in free ranging species. Currently, wildlife neoplasia is not only of interest to veterinarians but has also gained relevance in ecological research, particularly regarding the relationship between oncogenesis and environmental factors (2–4).

In New World monkeys, neoplasia has primarily been studied in species used as models for human disease, including *Callithrix*, *Saimiri*, *Aotus*, and occasionally *Alouatta*. Several types of neoplasia have been documented in *Alouatta* species. For example, a chondro-osteoblastic lipoma was reported in a seven-month-old mantled howler monkey (5), while pheochromocytoma (6), extrahepatic cholangiocarcinoma (7), and desmoplastic ameloblastoma (8) have been diagnosed in the black howler monkey (*Alouatta caraya*). A mediastinal T-cell lymphoma was identified in a brown howler monkey (*Alouatta guariba*) (9). In addition, a retrospective study of monkey skulls reported four skull masses classified as osteomas, although histologic confirmation was unavailable (10). Brain meningiomas were reported in two baboons (*Papio ursinus*) (11, 12) and in prosimians such as the collared brown lemur (*Eulemur collaris*) (13).

Overall, meningiomas appear to be rare spontaneous neoplasms in non-human primates, with very limited reports in monkeys (14). In contrast, they are among the most common primary intracranial tumors occurring in human adults (15) and in domestic species, such as cats and dogs (16).

## Case description

**Figure 1:**
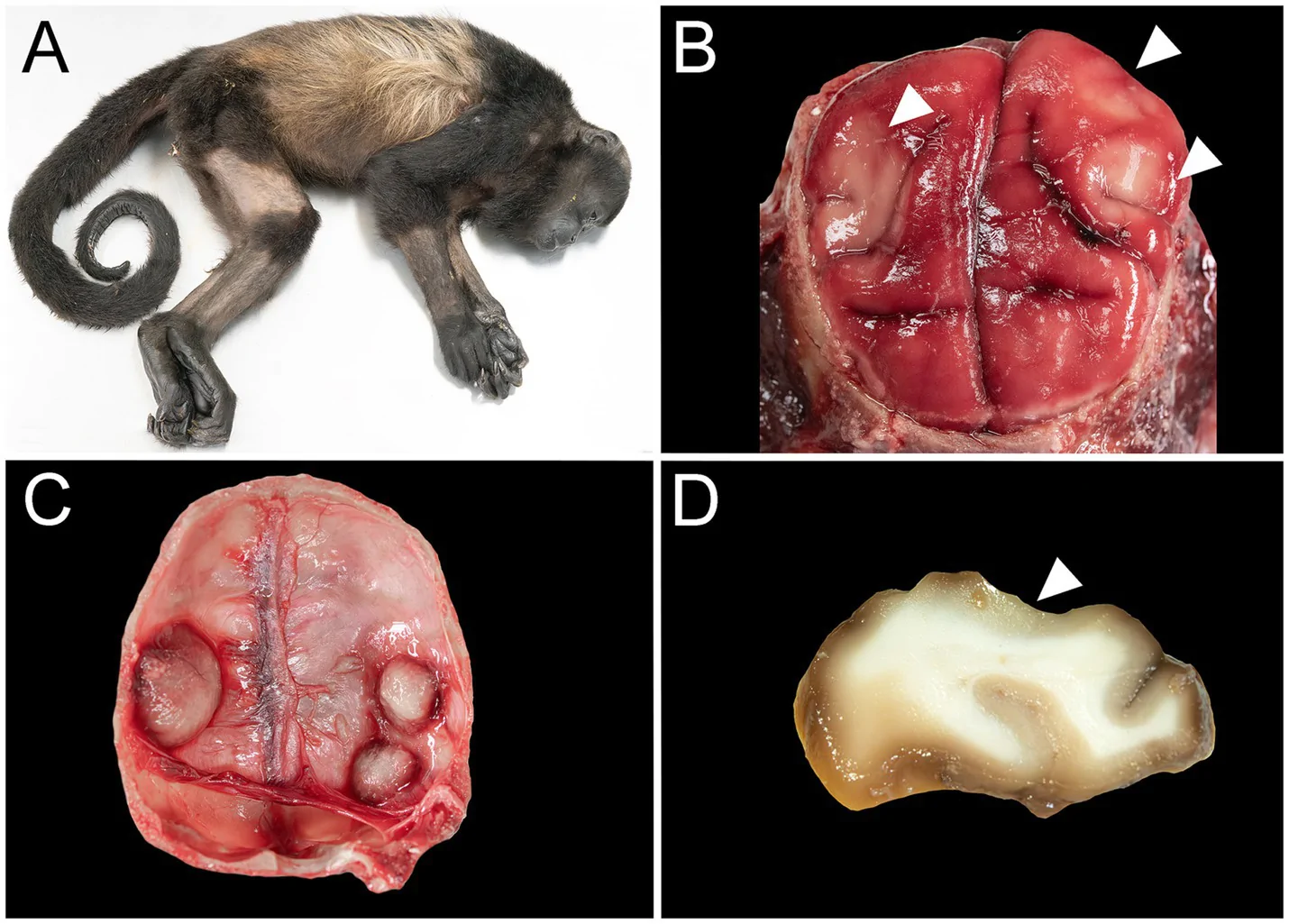
Necropsy findings. **(A)** Adult female mantled howler monkey (*Alouatta palliata*) before necropsy. **(B)** Cerebral cortex *in situ* showing three areas of cortical compression (arrowheads) caused by an intracranial meningioma. **(C)** Meningioma attached to the dura mater on the inner surface of the parietal bones. **(D)** Detailed view of cerebral cortical compression (arrowhead) caused by the intracranial meningioma.

An adult, female, freeranging, mantled howler monkey (Figure 1) was found conscious but non-ambulatory on a country road in Cuajiniquil, Costa Rica (10.03639°N, −85.52306°W). The animal was transported to the Rescate Wildlife Rescue Cente, Alajuela, Costa Rica for veterinary evaluation and further medical treatment. The animal was estimated to be older than 10 years based on its physical appearance and dental condition. At presentation, the animal was emaciated with a body weight of 2.8 kg. Neurological deficits were not detected; however, the patient exhibited severely depressed mentation and was hypoglycemic and hypothermic. The animal was rescued and euthanized approximately 3 h later due to its poor general condition and extensive soft tissue damage caused by myiasis. The carcass was frozen, and necropsy was performed 2 weeks later.

At necropsy, the animal was in poor body condition with no subcutaneous fat. The liver was congested, firm, and had dilated biliary ducts. The gallbladder contained numerous trematodes (~0.5 mm), most likely *Controrchis* sp. Upon opening the skull, three firm, gritty, white masses were found on the inner surface of the parietal bones (Figure 1), originating from the dura mater and compressing the cerebral cortex. These masses measured 0.5–1 cm in diameter (Figure 1). No other remarkable findings were observed during necropsy, including trauma-associated lesions.

**Figure 2:**
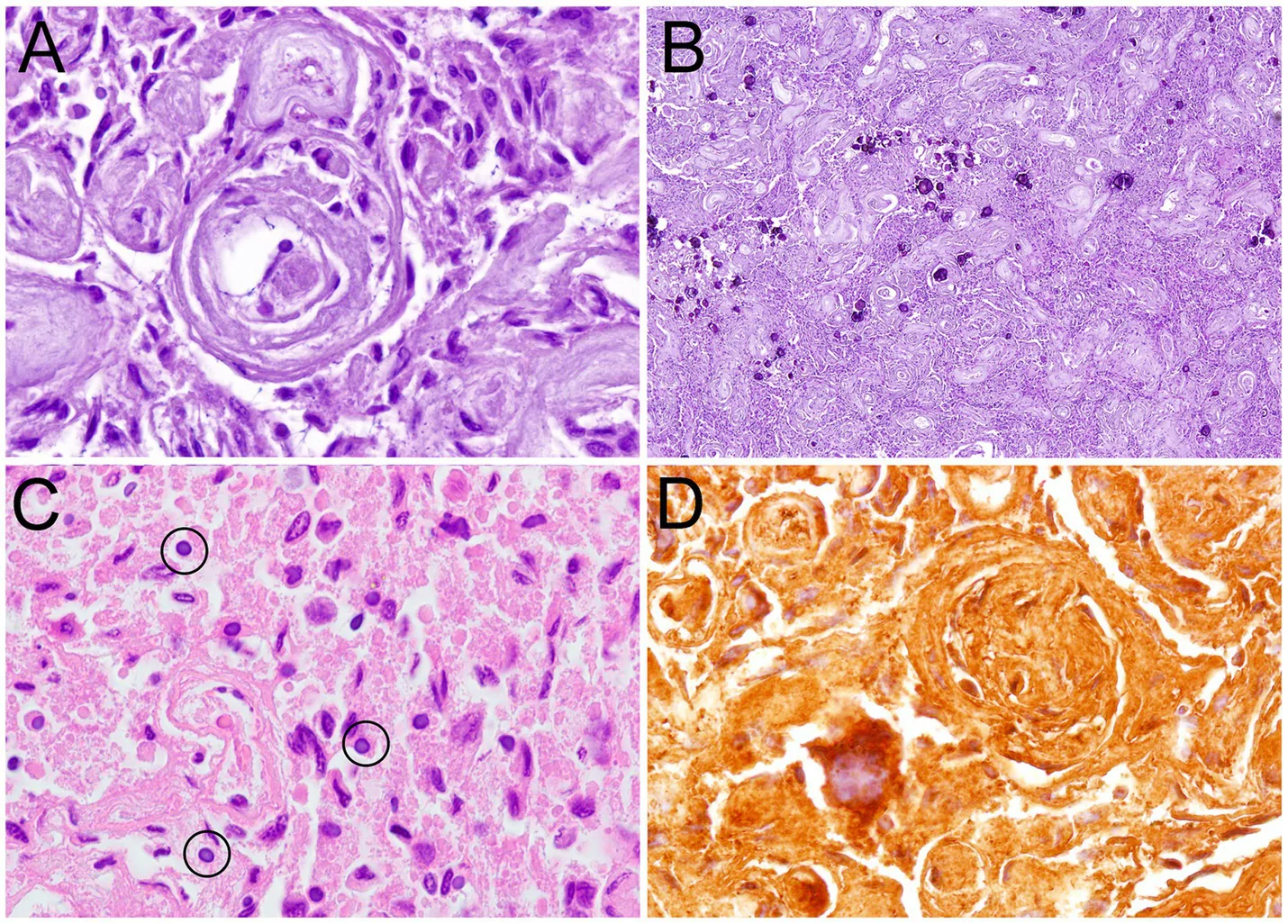
Histopathologic- and immunohistochemical staining **(A)** Hematoxylin and eosin (H&E) stained image showing whorled arrangement of neoplastic cells characteristic of a meningioma; 40x magnification. **(B)** Low-magnification view (4x) of H&E stained image showing whorled patterns and psammoma bodies within the neoplastic cell population. **(C)** H&E stained image showing intranuclear pseudo-inclusions in neoplastic cells; 60x magnification. **(D)** Immunohistochemical staining showing positive cytoplasmic labeling for S100 in neoplastic cells forming a whorl; 40x magnification.

Tissues were fixed in 10% buffered formalin and processed routinely for hematoxylin and eosin (H&E) staining. Microscopically, the masses consisted of a well circumscribed, encapsulated, and moderately cellular neoplastic cell population arising from the dura mater and compressing the cerebral cortex without infiltrating the leptomeninges. The cells were arranged in whorls (meningothelial pattern) and occasionally in interlacing streams (fibrous pattern), supported by a moderate amount of fibrocollagenous stroma. Two phenotypically distinct populations were present: the cells forming the whorls were medium-sized, spindeloid, with indistinct borders and moderate eosinophilic cytoplasm. Adjacent to the whorls, cells appeared polygonal with mild to moderate amounts of cytoplasm (Figure 1). Nuclei were centrally located, with dense chromatin and inconspicuous nucleoli; mitotic figures were rare (<1/10 HFP; Area = 1.6 mm^2^). Mild anisocytosis and anisokaryosis were observed. Occasional psammoma bodies and nuclear pseudo-inclusions were also present (Figure 2). Based on these histomorphologic features and anatomical location, the tumor was diagnosed as a transitional meningioma.

To further characterize the tumor, Immunohistochemistry (IHC) protocols and antibodies were standardized before immunoprofiling, using intestine (cytokeratins, vimentin and E-cadherin) and central nervous system tissues (S100) as positive controls from a different mantled howler monkey (Table 1). The antibody panel was selected based on markers previously validated in canine meningiomas (17). IHC staining was performed using the avidin–biotin complex method. Briefly: IHC was performed using the avidin–biotin complex method after optimization on normal intestinal and central nervous system tissues from another *A. palliata*. Following deparaffinization, antigen retrieval in citrate buffer (pH 6) was applied for all antibodies except S100, which required no pretreatment. Primary antibodies and dilutions are listed in Table 1. DAB was used as chromogen, and sections were counterstained with hematoxylin (18). Among the antibodies tested, S100 was the only marker that yielded positive staining in the tumor (Figure 2).

**Table 1:** Antibodies used for immunohistochemistry staining in normal tissue and meningioma.

Antibody	Target(s)	Clone type	Host	Manufacturer (Catalog No.)	Dilution
AE1/3	CK1–8, CK10, CK14–16, CK19	Monoclonal	Mouse	Dako (M3515)	1:200
MNF116	CK5, CK6, CK8, CK17, CK19	Monoclonal	Mouse	Dako (M0821)	1:1000
HMW	CK1, CK5, CK10, CK14	Monoclonal	Mouse	Dako (M0630)	1:500
CK-Pan	CK 48, 51, 52, 56, 58, 60, 68	Polyclonal	Rabbit	Novus Biologicals (NB600-579)	1:100
CK-Pol	Broad-spectrum cytokeratins	Polyclonal	Rabbit	Dako (Z0622)	1:500
Vimentin	Type III intermediate filament protein	Monoclonal	Mouse	Boehringer (1112457)	1:100
S100	Calcium-binding proteins	Polyclonal	Rabbit	Dako (Z0311)	1:2000
E-cadherin	E-cadherin	Monoclonal	Mouse	BD Transduction Laboratories (610181)	1:100

CK = cytokeratin.

## Discussion

Transitional meningiomas are generally considered benign, as they typically show slow growth and lack tissue infiltration (15), allowing the animal to gradually compensate for the increase in intracranial pressure (19). However, these compensatory mechanisms can eventually become overwhelmed, leading to compression of adjacent neural structures, and significant neurologic or behavioral alterations. In dogs, the most common clinical signs associated with meningiomas are seizures, menace response deficits, ataxia, abnormal mentation, and reduced postural reactions (20). Extrapolating these neurologic alterations to wildlife suggests reduced fitness, increased susceptibility to predation or accidents, and elevated mortality risk, as likely occurred in this case. Nonetheless, the impact of intracranial neoplasia on wildlife populations remains largely unexplored.

In the World Health Organization classification of central nervous system tumors in humans, meningiomas are divided into 15 subtypes (15). However, the histological classification of tumors of the nervous system in domestic animals categorizes meningiomas into three grades: grade I, grade II (atypical meningioma), and grade III (malignant meningioma). Each grade comprises several histological subtypes. Grade I includes the transitional (mixed), fibrous (fibroblastic), meningothelial, psammomatous, angiomatous, microcystic, and secretory subtypes. Grade II includes the atypical, chordoid, and clear cell subtypes. Grade III comprises the papillary and rhabdoid subtypes. The presence of nuclear pseudoinclusions, which are cytoplasmic invaginations within the nuclei, can be expected in domestic animals (21). This has been further described in rhabdoid meningiomas in dogs (22). In humans, it is a common diagnostic feature that is more associated with the meningothelial subtype (15).

Grading of meningiomas in human medicine relies on the mitotic index (mitotic figures per 10 HPF), brain invasion, morphologic subtype, anaplasia, and other features (15). Recently, a grading system was proposed for canine meningiomas (23, 24). Based on the currently available human and canine grading criteria, the current neoplasia in our study would most closely resemble a grade I meningioma.

Although this topic has only been briefly investigated in veterinary medicine, available data suggest that the molecular mechanisms underlying meningioma formation differ between humans and domestic species. In human medicine, more than 50% of meningiomas are associated with allelic loss in the neurofibromatosis type 2 (NF2) gene (15). The other major molecular mechanisms are mutations in the TRAF7, KLF4, AKT1, and SMO genes (25). In veterinary medicine, complete loss of NF2 has not been observed in dogs with meningioma (26). However, protein 4.1B and tumor suppressor lung cancer-1 (TSLC1) appear to play more important roles (27).

Immunohistochemical markers for meningioma in companion animals have been reviewed elsewhere (16). Overall, there is no single specific marker for the diagnosis of meningiomas across species, and marker positivity, such as cytokeratin AE1/3, laminin, claudin-1, glucose transporter 1, progesterone receptor, protein gene product 9.5, neuron-specific enolase (NSE), S100, and glial fibrillary acidic protein, seems to be species-dependent (16, 28, 29). In the present case, S100 positivity supports the diagnosis of a transitional meningioma. However, this marker is not consistently expressed in meningiomas and may also be positive in other intracranial neoplasms (16, 28, 29). Nonetheless, the histopathologic features seen in H&E staining are conclusive.

Regarding the negative staining toward the other antibodies, multiple reports describe heterogeneous or absent immunolabeling in meningiomas; for instance, cytokeratin KL1 staining in a group of human transitional meningiomas was negative (30). Other markers, such as Sox10, have been used in humans and appear to be more sensitive than S100 in the diagnosis of poorly differentiated meningiomas (31). Nevertheless, the Sox10 marker was recently tested in 10 canine meningiomas, all of which were negative (32). Positivity to vimentin was expected in the present case. Immunolabeling by this marker appears to be species-dependent, as variable expression has been reported among different taxa (16, 29, 30). Within the neoplastic tissue, there were only few vimentin-positive cells. Nonetheless, the vast majority of meningothelial and fibrous cells were negatively stained. Autolysis, freezing and/or prolonged formalin fixation may affect protein epitopes and influence IHC sensitivity. However, tissue preservation was considered acceptable, based on the quality of the H&E staining in the present case. It is well recognized that no single immunomarker is definitive for the diagnosis of meningioma. However, vimentin, E-cadherin, and CD34 are expected to be positive in canine meningiomas (16, 28), and epithelial membrane antigen (EMA) and claudin-1 are expected to be positive in human meningiomas (33). Despite this, histologic features and anatomical location remain the cornerstone for diagnostic confirmation.

In human medicine, known risk factors for meningioma include ionizing radiation, female sex, advanced age, and pesticide exposure. Comparable studies in veterinary or wildlife species are scarce. Anthropogenic pressure can lead to environmental contamination with potentially carcinogenic pollutants, including agrochemicals (1). The extent of this animal’s exposure to such agents is unknown, but increased surveillance for neoplastic diseases in wildlife is warranted to assess potential environmental impacts.

This report documents a spontaneous transitional meningioma in a free ranging mantled howler monkey. To the authors’ knowledge, this is the first reported case of this neoplasm in a neotropical non-human primate. Documentation of neoplasia in free ranging wildlife remains limited, and reports such as this one contribute to the growing field of wildlife oncopathology. In non-human primates, reports of neoplasia remain rare, particularly in free ranging individuals; most available data are derived from captive animals or isolated case reports. The present case therefore highlights the occurrence of such tumors outside managed populations and advances our understanding of disease occurrence in these populations, particularly in view of rising anthropogenic pressures.

## Data Availability

The original contributions presented in the study are included in the article/supplementary material, further inquiries can be directed to the corresponding author.

## References

[1] Pesavento PA Agnew D Keel MK Woolard KD. Cancer in wildlife: patterns of emergence. Nat Rev Cancer. (2018) 18:q17–q45. doi: 10.1038/s41568-018-0045-0, 30116020

[2] Dujon AM Ujvari B Tissot S Meliani J Rieu O Stepanskyy N . The complex effects of modern oncogenic environments on the fitness, evolution and conservation of wildlife species. Evol Appl. (2024) 17:e13763. doi: 10.1111/eva.13763, 39100750 PMC11294924

[3] Giraudeau M Sepp T Ujvari B Ewald PW Thomas F. Human activities might influence oncogenic processes in wild animal populations. Nat Ecol Evol. (2018) 2:1065–70. doi: 10.1038/s41559-018-0558-7, 29784981

[4] Giraudeau M Vincze O Dupont SM Sepp T Baines C Lemaitre JF . Approaches and methods to study wildlife cancer. J Anim Ecol. (2024) 93:1410–28. doi: 10.1111/1365-2656.14144, 39189422 PMC11745198

[5] Berrocal A Vail K Gonzalez O Shivanna V Dick EJ Jr Watanabe TTN . Lingual neoplasia in nonhuman primates: description of five cases and a literature review. J Med Primatol. (2024) 53:e12725. doi: 10.1111/jmp.12725, 39034453 PMC13098483

[6] Juan-Sallés C Ramos-Vara J Garner M. Pheochromocytoma in six New World primates. Vet Pathol. (2009) 46:662–6. doi: 10.1354/vp.08-VP-0275-J-BC, 19276065

[7] De Santi M Do Couto C Montanhim GL Moraes PC Bertolo PHL Vasconcelos R d O . Extrahepatic cholangiocarcinoma in an adult black howler monkey (Primates: Atelidae). Braz J Vet Med. (2019) 41:e101519–e101519.

[8] Luna CA Natalini MB Repetto C Sanchez Gavier F Cuaranta P Kowalewski MM. Desmoplastic ameloblastoma in a specimen of *Alouatta caraya* (Primates, Atelidae) from northeastern Argentina. J Med Primatol. (2025) 54:e70015. doi: 10.1111/jmp.70015, 40181640

[9] de Souza AJS da Silva VL de Sá LRM. Mediastinal T-cell lymphoma in a free-ranging brown howler monkey (*Alouatta guariba clamitans*). J Med Primatol. (2024) 53:e12663. doi: 10.1111/jmp.12663, 37496256

[10] DeGusta D Milton K. Skeletal pathologies in a population of *Alouatta palliata*: behavioral, ecological, and evolutionary implications. Int J Primatol. (1998) 19:615–50. doi: 10.1023/a:1020372825031

[11] Oliveira F Porter B Dick E Jr Hubbard G. Intracranial meningioma in a baboon (Papio spp.). J Comp Pathol. (2011) 145:414–8. doi: 10.1016/j.jcpa.2011.03.006, 21570692 PMC3530411

[12] McConnell E. Basson P. De Vos V. Myers B. J. Kuntz R. A survey of diseases among 100 free rangingbaboons (*Papio ursinus*) from the Kruger National Park. (1974). 4469830

[13] Remick A Van Wettere A Williams C. Neoplasia in prosimians: case series from a captive prosimian population and literature review. Vet Pathol. (2009) 46:746–72. doi: 10.1354/vp.08-VP-0154-R-FL, 19276064

[14] Miller AD Kirejczyk SGM. "Nonhuman primate neoplasia". In: Kondova-Perseng I Mansfield KG Miller AD, editors. Atlas of Diagnostic Pathology in Nonhuman Primates [Internet]. Cham: Springer International Publishing (2024). p. 229–54.

[15] Board WC of TE. Central Nervous System Tumours [Internet]. (n.d.). Available online at: https://publications.iarc.who.int/Book-And-Report-Series/Who-Classification-Of-Tumours/Central-Nervous-System-Tumours-2021

[16] Rissi DR Miller AD Demeter EA Church ME Koehler JW. Diagnostic immunohistochemistry of primary and secondary central nervous system neoplasms of dogs and cats. J Vet Diagn Invest. (2024) 36:153–68. doi: 10.1177/10406387231221858, 38234003 PMC10929637

[17] Montoliu P Añor S Vidal E Pumarola M. Histological and immunohistochemical study of 30 cases of canine meningioma. J Comp Pathol. (2006) 135:200–7. doi: 10.1016/j.jcpa.2006.06.006, 17049358

[18] Gregor KM Lakemeyer J IJsseldijk LL Siebert U Wohlsein P. Spontaneous neoplasms in harbour porpoises *Phocoena phocoena*. Dis Aquat Org. (2022) 149:145–54. doi: 10.3354/dao03670, 35735234

[19] Miller AD Miller CR Rossmeisl JH. Canine primary intracranial cancer: a clinicopathologic and comparative review of glioma, meningioma, and choroid plexus tumors. Front Oncol. (2019) 9. doi: 10.3389/fonc.2019.01151, 31788444 PMC6856054

[20] Forward AK Volk HA Cherubini GB Harcourt-Brown T Plessas IN Garosi L . Clinical presentation, diagnostic findings and outcome of dogs undergoing surgical resection for intracranial meningioma: 101 dogs. BMC Vet Res. (2022) 18:88. doi: 10.1186/s12917-022-03182-y, 35249530 PMC8900440

[21] Jubb, Kennedy, and Palmer’s Pathology of Domestic Animals: Volume 1 [Internet]. (2025). Available online at: https://shop.elsevier.com/books/jubb-kennedy-and-palmers-pathology-of-domestic-animals-volume-1/maxie/978-0-443-12071-8

[22] Tabaran AF Armien AG Pluhar GE O’Sullivan MG. Meningioma with rhabdoid features: pathologic findings in dogs. Vet Pathol. (2022) 59:759–67. doi: 10.1177/03009858221100436, 35674149

[23] Belluco S Marano G Lurier T Avallone G Brachelente C Di Palma S . Standardization of canine meningioma grading: validation of new guidelines for reproducible histopathologic criteria. Vet Comp Oncol. (2023) 21:685–99. doi: 10.1111/vco.12932, 37635372

[24] Belluco S Marano G Baiker K Beineke A Oevermann A Seehusen F . Standardisation of canine meningioma grading: inter-observer agreement and recommendations for reproducible histopathologic criteria. Vet Comp Oncol. (2022) 20:509–20. doi: 10.1111/vco.12802, 35066998

[25] Clark VE Erson-Omay EZ Serin A Yin J Cotney J Özduman K . Genomic analysis of non-NF2 meningiomas reveals mutations in TRAF7, KLF4, AKT1, and SMO. Science. (2013) 339:1077–80. doi: 10.1126/science.1233009, 23348505 PMC4808587

[26] Thomas R Duke SE Wang HJ Breen TE Higgins RJ Linder KE . ‘Putting our heads together’: insights into genomic conservation between human and canine intracranial tumors. J Neuro-Oncol. (2009) 94:333–49. doi: 10.1007/s11060-009-9877-5, 19333554 PMC3225023

[27] Dickinson PJ Surace EI Cambell M Higgins RJ Leutenegger CM Bollen AW . Expression of the tumor suppressor genes NF2, 4.1B, and TSLC1 in canine meningiomas. Vet Pathol. (2009) 46:884–92. doi: 10.1354/vp.08-VP-0251-D-FL, 19429976

[28] Ramos-Vara JA Miller MA Gilbreath E Patterson JS. Immunohistochemical detection of CD34, E-cadherin, Claudin-1, glucose transporter 1, laminin, and protein gene product 9.5 in 28 canine and 8 feline meningiomas. Vet Pathol. (2010) 47:725–37. doi: 10.1177/0300985810364528, 20403881

[29] Saito R Chambers JK Kishimoto TE Uchida K. Pathological and immunohistochemical features of 45 cases of feline meningioma. J Vet Med Sci. (2021) 83:1219–24. doi: 10.1292/jvms.21-0258, 34162785 PMC8437717

[30] Artlich A Schmidt D. Immunohistochemical profile of meningiomas and their histological subtypes. Hum Pathol. (1990) 21:843–9. doi: 10.1016/0046-8177(90)90054-9, 1696924

[31] Ng J Celebre A Munoz DG Keith JL Karamchandani JR. Sox10 is superior to S100 in the diagnosis of meningioma. Appl Immunohistochem Mol Morphol. (2015) 23:215–9. doi: 10.1097/PAI.0000000000000072, 25265429

[32] Nelissen S Miller AD. Assessment of SOX10 expression in 437 canine neoplasms of different embryologic origins. Vet Pathol. (2024) 61:704–11. doi: 10.1177/03009858241231562, 38366813

[33] Hahn HP Bundock EA Hornick JL. Immunohistochemical staining for Claudin-1 can help distinguish meningiomas from histologic mimics. Am J Clin Pathol. (2006) 125:203–8. doi: 10.1309/G659FVVBMG7U4RPQ, 16393681

